# Trends and Innovations of Simulation for Twenty First Century Medical Education

**DOI:** 10.3389/fpubh.2022.619769

**Published:** 2022-03-03

**Authors:** Eduardo Herrera-Aliaga, Lisbell D. Estrada

**Affiliations:** Faculty of Health Sciences, Universidad Bernardo O'Higgins, Santiago, Chile

**Keywords:** simulation, healthcare, competences, COVID-19, medical education, public health

## Abstract

In the last two decades there has been an enormous growth in the use of clinical simulation. This teaching-learning methodology is currently the main tool used in the training of healthcare professionals. Clinical simulation is in tune with new paradigms in education and is consistent with educational theories that support the use of experiential learning. It promotes the development of psychomotor skills and strengthens executive functions. This pedagogical approach can be applied in many healthcare topics and is particularly relevant in the context of restricted access to clinical settings. This is particularly relevant considering the current crisis caused by the COVID-19 pandemic, or when trying to reduce the frequency of accidents attributed to errors in clinical practice. This mini-review provides an overview of the current literature on healthcare simulation methods, as well as prospects for education and public health benefits. A literature search was conducted in order to find the most current trends and state of the art in medical education simulation. Presently, there are many areas of application for this methodology and new areas are constantly being explored. It is concluded that medical education simulation has a solid theoretical basis and wide application in the training of health professionals at present. In addition, it is consolidated as an unavoidable methodology both in undergraduate curricula and in continuing medical education. A promising scenario for medical education simulation is envisaged in the future, hand in hand with the development of technological advances.

## Introduction

Medical education has experienced rapid changes worldwide (1, 2) in line with all present challenges. These changes emerged as the product of various problems, including the changing needs of the population and the multiple scientific and technological advances generated by the evidence-based accumulation of medical knowledge. The changing world of medical education, and the consolidation of new educational paradigms, demand the incorporation of innovative strategies (3). Medical education is at the center of these phenomena, since it must use the best educational strategies to transform inexperienced students into competent professionals. This permanent struggle has contributed to the emergence of new and innovative methodologies for teaching, learning and assessment.

Clinical simulation is an innovative methodology for medical education and has been developing rapidly in recent years. Gaba (4) defined clinical simulation as: “A technique, not a technology, to replace or amplify real experiences with guided experiences that evoke or replicate substantial aspects of the real world in a fully interactive manner”. Simulation allows teaching through guided experiences in safe contexts, facilitating adequate learning and standardized assessment of the skills necessary to face a changing world.

Herein, we summarized the history and context of clinical simulation, describing the current state of the art in this area, and suggesting future directions. This review aims to contribute to the formation of a new generation of health educators and professionals that would ultimately benefit patient treatment and public health. We searched PubMed, Web of Science and additional databases for publications, including original articles, systematic reviews and meta-analyses regarding recent evidence on clinical simulation and new developments in this area.

## The Past and Present of Simulation

Simulation began before the appearance of man because it is evident in nature, where mimicry is common. According to Jean Baudrillard, when mimicry occurs, it is possible to supplant reality. In 1911, Hartford Hospital used the first full-body simulator for nursing skills training and called it “Ms. Chase” (5, 6). The appearance of simulation is of capital importance in aviation training. Actually, the invention of the “Link Trainer” flight simulator was particularly useful for training thousands of pilots during World War II (7). Currently, the aviation industry uses simulation as a fundamental strategy for training its personnel, helping to build high safety standards (8). A simulator is more effective in training some piloting maneuvers and “no one could imagine using an aircraft to train today” (9). Observing at the practices used to minimize error in aviation can provide new options that can be used in health to reduce medical errors (10). Since the middle of the last century, several simulators have been developed for medical education, such as the Resusci Anne™, SimOne™, Noelle™, and SimMan™, which have increased the fidelity of the simulated scenarios and made them more realistic.

Simulation centers are located all over the world, including simulation hospitals or virtual hospitals that have the same equipment found in actual hospitals. This allows students to be trained and to requalify professionals who are already interacting with patients. Along this line, simulation is one of the key elements used in continuing education (11), aiming to maintain and improve previously acquired skills in conditions similar to the ones present in real environments. This approach has significant advantages over other methodologies because it allows the acquisition of skills in “knowing,” “knowing how to do,” and “knowing how to be” (12).

Scenarios used in simulation can be predictable, standardized, controlled, safe and reproducible. The, simulation allows training of skills needed for situations that occur infrequently, such as the management of cardio-respiratory arrests. The simulation scenarios are also repeatable, until an optimal degree of training can be achieved. This allows good performance in real clinical practice when an equivalent situation is faced. Repetition of the skill with adequate feedback allows for a high degree of training. Simulations may not be perfect and might not represent reality at its finest. Thus, it is relevant to generate a “fiction contract” and communicate and guide the student, so that this “lack of reality” does not affect future performance. Scenarios should be subject to constant review to ensure they are up to date and valid. One limitation of this methodology is the duration of a scenario, which is not equal to a real-life event. Therefore, the instructor's guide, the brief, and debriefing are key processes: the “transfer” of what the student has learned and its application to a real (and possibly stressful) situation.

The simulation provides immersive environments. Multisensory learning is possible with this modality, because intense emotions are involved in contexts of psychological safety and effective feedback, all of which allow long-term learning. This improves the efficiency of educational systems in relation to costs and training times. Engaging emotions could facilitate long-term learning, although the association between both aspects is not completely clear (13). Learning through emotion is advantageous to the acquisition of skills compared to other methodologies (14).

Currently there are several types of simulation ranging from training with part simulators for specific skills to training multiple and complex skills in immersive environments using strategies such as virtual reality, surgical simulation (15) and the use of standardized patients.

## The Typology of Simulation

The typologies allow adapting the simulation to different curricular levels and incorporate related variables, such as the competences to be achieved and resources, available. The simulations were initially classified as low-, medium- or high-fidelity. Fidelity is the degree to which the simulation mimics reality: the higher the fidelity, the greater the sense of realism. Low fidelity simulations are used for training on specific skills and in novice students who do not visualize the context. In that sense, the simulation should be based on small tasks from simple to complex (16). At medium fidelity, additional elements are added and some interaction with environmental “noise” or simulator signals are possible. In high-fidelity simulations, immersion is complete, and the setting of the scenarios are complex. High-fidelity simulations are useful for participants of advanced or competent levels who have already passed the training in parts. These simulations are used for training in more complex skills, such as clinical skills, communication, decision making, crisis resource management, and critical thinking (17).

There are also other typologies. Ziv et al. (18) proposed five simulation categories that are contextualized according to “tools.” These categories are: (i) low-tech simulators or part-task trainers, (ii) simulated/standardized patients, (iii) screen-based computer simulators, (iv) complex task coaches, and (v) realistic patient simulators. Gaba (4) proposed a classification according to the available technologies: (i) verbal (role play), (ii) standardized patients (actors), (iii) part-task instructors (physical; virtual reality), (iv) computer patient (screen-based, virtual world, etc.), and (v) electronic patient (replica of clinical site; mannequin-based; full virtual reality). Alinier (19) proposed a six-level classification system that ranges from the simplest simulations (level 0) to the most complex (level 5), which allows choosing the level according to the skills, the necessary resources, among other variables. The Boston Children's Simulator Program provides a useful guide to planning scenarios with five areas of increasing complexity: Zone 0: basic technical skills training; Zone 1: training of clinical competences; Zone 2: contextualized skills training in more complex/virtual environments, with a focus on clinical decisions; Zone 3: team building and multidisciplinary teams; and Zone 4: real life events.

## Theories Applicable to Simulation

Adults have a plethora of previous experiences, they value that the learning of values is relevant and applicable to concrete situations (20), prefer problem solving, and possess internal motivation. The educational theories applicable to simulation are the theories described by Vytgosky, Kolb, Dreyfus and Drefyus, Posner, Schon, Bandura and Ericcson, among others. Vytgosky describes the concept of “Zone of Proximate Development,” which establishes the progress that a student must have (21). David Kolb describes “Experiential Learning” (20). Dreyfus and Dreyfus describe the existence of skill acquisition levels, from novice to expert (16). Posner establishes consecutive phases for the acquisition of skills (21). For Schon, the reflection on the practice is relevant (21). Bandura presented his theory of self-efficacy, which consists of the perceived capacity of the person to carry out a task. In this way, the greater the perception of self-efficacy, the higher the success rate (22). Ericcson states that the repetitive practice of an activity leads to the acquisition of skills (23). The cognitive load theory establishes that working memory has a limited capacity, and that excess cognitive load is counterproductive for learning (24). The theories described above provide a basis for the concrete and practical application of simulation, how learning occurs, how to insert simulation into the curriculum and how to graduate the complexity of the scenarios.

Simulation is an “active” learning methodology, because it involves the participation and observable actions of the student. In the adult student, active participation increases the effectiveness of learning (20). In simulations, the student interacts with basic or complex simulators, or with standardized patients (which simulate pathologies and allow communication or anamnesis), or they can interact with other students or health professionals, allowing teamwork.

In deliberate practice, the skill is repeated in relation to predefined objectives to achieve a high degree of mastery. The key aspects are setting clear, pre-set goals and then making the student to repeat the tasks to improve specific skills. Students receive feedback on their performance. Rapid Cycle Deliberate Practice (RCDP), is used for training specific skills, and involves repetitive practice of increasing complexity, with an emphasis on real-time error correction (25).

The simulation must be based on adequate teaching practices to reach the desired impact (26, 27). These are: having defined goals, giving effective feedback, offering repetitive practices, integrating simulation into the curriculum, having different levels of training and multiple learning strategies, offering clinical variation, having controlled environments, and having individualized learning. Besides, the use of standardized checklists is key to facilitate training and to propitiate appropriate transfer to actual clinical situations (28).

## The Importance of Debriefing in Medical Simulation

The debriefing corresponds to the systematic reflection on experiences during the simulation (29). Debriefing transforms the experience into learning through reflection (30). According to Kolb (20), when people experience situations (concrete experience), they must reflect on the lived situation, then form abstract concepts and finally test what they have learned in the new situations. The debriefing allows re-examination of the experience (31), and is carried out according to a pre-defined method with certain rules. The debriefing takes place immediately after the experience and is not only applicable to simulation, but also to real clinical experiences. Therefore, reflection is essential, and when carried out in a systematic way, successful results are obtained.

Various types of debriefing are described in the literature. Typically, they are carried out in three phases: Reaction, Analysis and Summary (29). Steinwachs also describes 3 phases: Description, Analogy/Analysis and Application (32). The Center for Medical Simulation (33) describes the three phases as: Reactions Phase, Comprehension Phase and Application Phase. The “D.E.B.R.I.E.F.” model establishes a mnemonic to guide debriefing: *D*efine rules, Explain learning objectives, Bench marks for performance, Review what should happen, Identify what happened, Examine why, and Finalize/formalize learning. In the “3D Model,” three phases are also proposed: *D*efusing, Discovering and Deepening (29, 34) and the “GAS method” proposes: *G*ather, Analyze and Summarize (29). Recently a type of debriefing that we know as CORE Debriefing has been introduced, which has 4 phases: *C*ompression, Observation, Reflection and Exchange.

The “debriefing with good judgment” approach is a form of debriefing in which the instructor's goal is to understand the students' point of view, to determine how their mental models produce actions that translate into their performance (double loop). The instructor assumes a quota of curiosity and can issue his judgment in a respectful and constructive way- from the observation perspective of the specific events that occurred during the scenario (“good judgment”) (33). In this style of debriefing, the thought processes of the students are crucial: it is important to elucidate the reason behind one decision or another, exploring the mental models of their choice, beyond the students' performance (33).

## Current Trends and Innovation in Simulation Education

Virtual reality has posited a significant increase in simulation education; however, its effectiveness is not overwhelming (35–40). Simulation has a positive impact on various training areas, such as work in clinical teams (41), improvement in surgical skills (42–47), in ophthalmology (48), in specific health techniques (49–52), critical care (53), pediatrics (54), resuscitation (55), medical emergencies (56), microsurgery (57), anesthesia (58), and nursing (59–61). Training through “Low-Dose and High Frequency” settings can be effective for CPR training (62, 63). The use of standardized patients is common in simulation and has a positive impact on interprofessional education (64). Although its use is frequent in continuing education, there are not many reports describing its effectiveness (65). The interprofessional simulation has a good perception according to some reports (66). Regarding the impact of simulation training on the quality of patient care (translational studies), research must be developed further and grow in number. Currently, there is no evidence to elucidate a strong impact of simulation education on this specific matter (44, 45, 50, 58, 67–70).

There is a wide field of development and validation of new simulation areas. Importantly, this is increasingly being incorporated into the education curriculum. *In situ* simulation is gaining importance for training in real environments and to encourage teamwork (71–74), which must be contrasted with its real effectiveness in certain contexts (73, 75). Surgical simulation emerges as an important development pole (76) while its use in previously unexplored areas such as psychiatry gains relevance (77). The validation of simulation programs is increasingly important, as well as the knowledge of how it impacts patient care (76). Simulation allows for the evaluation of competences. These mechanisms grow in validity and reliability and methods such as OSCE, OSATS, mini-CEX, and DOPS are consolidated. It is important to note that these assessments synchronize with teaching methods and that they should be properly integrated into the curriculum (78). Telesimulation has taken an important place, especially after the COVID-19 pandemic. Specifically, it consists of linking an instructor and a student who are geographically distant through the Internet. It is estimated that this modality has a positive impact on the acquisition of skills in various environments (79, 80); a simulation center or a clinical center with *ad-hoc* technology can provide distance training to centers with less technology (68, 81), and where distance is an impediment. This strategy allows remote evaluation and feedback (68, 80). Although there is a sustained increase in systematic reviews and meta-analyzes in relation to simulation, there is still work pending in terms of improving the methodological designs of the investigations.

## Importance of Simulation Education During the COVID-19 Pandemic

Universities worldwide announced immediate closures to prevent the spread of the virus, allowing their students to complete the semester remotely (82). This crisis imposed the need for virtual learning. To minimize disruption to teaching and assessment, some universities have replaced face-to-face classes with virtual ones. Unfortunately, this outbreak widens the gap between countries with technologies and support compatible with online learning and those the lack of these technological resources. Even at the same university, and in the same class, many students suffer from unequal access or lack digital connections (83). Maintaining performance standards and quality assurance are unprecedented challenges in pandemic conditions for most universities.

Simulation has become an important tool in meeting the challenges posed by COVID-19 (84). The simulation was necessary for the training and requalification of clinical teams in the face of the pandemic (“first line”), for example in crisis resource management, orotracheal intubation, mechanical ventilation, and the use of personal protective equipment (85).

Undergraduate education was affected by the inability to attend classes in person (80, 86). Keeping students engaged and up to date with the curriculum is especially challenging when they must learn practical subjects remotely. In our experience, a live streaming classroom using HD quality cameras, improved interaction and ensured remote participation of students to the same standard as face-to-face classes.

Virtual simulations increased, with successful implementation (87), and their impact is under evaluation. Using simulation-based platforms, it is possible to perform case-based scenarios online and run virtual OSCEs from anywhere in real-time. The virtual OSCE could offer a suitable platforms to address evaluation options (88, 89). Besides, this type of simulation allows the promotion of skills for decision-making in healthcare.

## Conclusion

Clinical simulation has made important contributions and is currently a valid and increasingly common option for medical education. Its multiple strategies make this approach useful to meet or overcome multiple challenges (90). In this way, simulation can be transformed into a powerful strategy to appropriately train health professionals to effectively address the challenges of today's changing world.

A positive impact of simulations in the training of graduates working in clinical environments is to reduce the risks by allowing professionals to prepare and anticipate complex clinical situations (17). A recent systematic review shows that *in situ* training improves patient outcomes, such as reduction in cardiac arrest rates or increase in incidence reporting rates (91). Still, few articles focus on patient outcomes as a measure of improved clinical competency, while most studies center on the skill's progression (91). Learning through simulation is frequently used in undergraduate degree curricula. Shortly, the simulation will be mandatory before confronting the patient. Likewise, real clinical experiences will give way to more simulated practical situations.

Simulation can be used in both undergraduate and postgraduate education, from simple situations such as suture training or an IV puncture, to medical interview training, or difficult situations such as emergencies or crisis resource management. The systematic implementation of simulation has advantages over other methodologies because it allows for greater efficiency in the educational process and also brings the clinical reality closer to the trainee without putting a patient's health at risk.

Simulation is a fundamental strategy for current and future challenges in medical education. The simulation has multiple strategies based on educational theories that allow effective learning. It should be convenient that all health professional training institutions adopt the simulation strategy and that health centers incorporate education through simulation for the continuous training of their professionals in interdisciplinary contexts. Systematic simulation training programs should be conducted to incorporate best practices. Finally, it is very convenient for health centers to train their teams frequently, for example, on a monthly basis incorporating deliberate practice, feedback and debriefing.

## Author Contributions

EH-A wrote the manuscript. LE wrote and edited the manuscript. All authors contributed to the article and approved the submitted version.

## Funding

This work was supported by Educational Research grant UBO/VVCMEI 20208.

## Conflict of Interest

The authors declare that the research was conducted in the absence of any commercial or financial relationships that could be construed as a potential conflict of interest.

## Publisher's Note

All claims expressed in this article are solely those of the authors and do not necessarily represent those of their affiliated organizations, or those of the publisher, the editors and the reviewers. Any product that may be evaluated in this article, or claim that may be made by its manufacturer, is not guaranteed or endorsed by the publisher.
